# On Students’ Willingness to Use Online Learning: A Privacy Calculus Theory Approach

**DOI:** 10.3389/fpsyg.2022.880261

**Published:** 2022-06-13

**Authors:** Xinyu Jiang, Tiong-Thye Goh, Mengjun Liu

**Affiliations:** ^1^School of Education, Hubei University, Wuhan, China; ^2^School of Information Management, Victoria University of Wellington, Wellington, New Zealand

**Keywords:** online learning, privacy calculus theory, benefit perception, risk perception, trust perception

## Abstract

Online learning platforms frequently collect and store learners’ data to personalize content and improve learning analytics, but this also increases the likelihood of privacy breaches which may reduce learners’ willingness to use online learning. This study aims to examine how perceptions of benefits, privacy, risk, and trust affect students’ willingness to use online learning. We used the Privacy Calculus Theory as a theoretical framework for this study. To test the model, we surveyed 203 undergraduate students who used online learning. The results of the AMOS analysis revealed that students’ risk perception has a significant negative effect on their willingness to use online learning, while their benefit perception and trust perception have positive effects. Furthermore, the study found that improved trust can reduce perceived risk and improve willingness to use online learning. Interestingly, privacy perception is not a significant predictor of students’ willingness to use online learning, although it is a high concern factor. Discussion and conclusion are discussed at the end.

## Introduction

Since the COVID-19 pandemic started, online learning has largely replaced face-to-face instruction and has developed into a viable alternative to delivering instruction at all levels of education. Online learning platforms are advantageous in delivering rich, comprehensive, and personalized content to individuals by profiling learners’ personal and behavioral data. While personalization can improve adoption, passive or active collection and storage of personal data increase the risk of data breaches. Because large amounts of confidential data are more attractive to hackers. Studies conducted by Bongiovanni (2019) and Osterman (2021) suggested that universities were not adequately protecting their computer assets. For instance, in May 2018, the security vulnerability of NetID, an online service system of the University of Vermont, compromised the personal data security of 37,000 current and former faculty members and students (Bongiovanni, 2019). In May 2019, the internal database of the Georgia Institute of Technology was attacked, resulting in the breach of 1.3 million data (Chapman, 2019). Most recently, a Spanish e-learning platform discovered a data breach affecting 150,000 users across the globe. In addition to personally identifiable data, students’ account details including the courses they have taken, account user IDs, their evaluation scores, and certificates of completion were compromised (DataBreach, 2020). In March 2020, Zoom, an application for online videoconferencing which was widely used as a “cloud classroom” amid the COVID-19 pandemic, suffered frequent VTC hijacks (also known as “Zoom-bombing”). Intruders gained access to online classrooms by stealing information from online conferences, posing a threat to the security of students’ data and learning information (Setera, 2020). While the pandemic has brought opportunities for online learning, it has also brought challenges. Increasing the security requirements for online learning is necessary and should continue after the pandemic.

Online learning is defined as a learning experience through the internet in a synchronous or asynchronous environment where students engage with instructors and fellow students at a time of their convenience (Singh and Thurman, 2019). In the online learning process, data is generated through the interaction between users and tools or platforms. Data can provide insight into behavior patterns and facilitate behavioral improvement. If effectively utilized, online learning providers can tailor personalized learning services to meet student’s educational needs; students, and educators can get the information they need to make decisions and create opportunities for student success. These are important reasons for students to engage in online learning. Increasingly, there is more data being collected and exchanged due to the widespread use of personal networks and devices accessing online learning. However, many educational institutions have not been adequately prepared to deal with sophisticated cybersecurity threats (Osterman, 2021). In addition to lacking sufficient human resources and financial resources to manage network security, online learning providers also lack sufficient knowledge of network security principles and defenses. Due to the COVID-19 pandemic, many institutions have accelerated the transformation of the person learning to online learning. Online learning environments are therefore subject to unprecedented security risks (EDUCAUSE, 2021).

Increasing privacy issues limit users’ trust in the online learning environment and the degree of information disclosure (Anwar, 2021). Students’ perception and awareness of security and how they respond to vulnerability will affect their motivation to learn, which in turn affects the effectiveness of online learning and hence their adoption behavior (Ali and Zafar, 2017). Therefore, the present study developed a conceptual model that considers how issues of data security and privacy, compared with the benefits of online learning, will affect students’ willingness to use online learning.

The term privacy calculus refers to the calculus of “human behavior.” Calculus refers to the cognitive trade-off among situational constraints which govern the decision-making process of individuals to decide whether to disclose personal information (Laufer and Wolfe, 1977). The principal components in privacy calculus are the perceived benefits and perceived risks. The privacy calculus theory implies that human agents act in a way to maximize benefits and minimize risks. Using the framework of privacy calculus can explain the joint influence of perceived benefits and risks on the concept of privacy and privacy protection behavior. Perceived benefits refer to the value acquisition that users perceive when information is disclosed, and perceived risks refer to potential losses that users perceive when information is disclosed. Hence the stronger the perceived benefits, the higher the possibility of information disclosure; when people worry about the potential risks, the less likely they disclose private information. Perceived benefits include social support, enjoyment, customized information, time and cost-saving, or monetary rewards. Perceived risks generally refer to the privacy and security concerns of online users, including identity theft, damaged reputation, or loss of control. Although the specific benefits and risks factors differ from study to study, overall findings supported the central point of privacy calculus theory (Smith et al., 2011). For example, in the environment of e-commerce and social networking, benefits such as personalization and money had a positive impact on information disclosure (Sun et al., 2019; Koh et al., 2020). Concerns about privacy issues have been found to have a negative relationship with users’ willingness to provide information and adoption of online services (Kim et al., 2019). The users may even choose to abandon the use and conduct personal privacy protection. In addition, users who are more concerned about privacy had stricter privacy settings for their data (Jozani et al., 2020).

Based on the privacy calculus theory, this manuscript proposes that students assess the perceived benefits and perceived risks of online learning to determine their adoption. Specifically, the greater the perceived benefits in online learning are more likely to induce learners to disclose personal information and use, while greater concern to information privacy and the perceived risks of privacy breaches will negatively impact learners’ willingness to use online learning. According to information system theories, money, convenience (Hann et al., 2007), efficiency (Krasnova et al., 2010), personalized functions (Zhu et al., 2016), and other factors will motivate individuals to use online services. Likewise, the benefits of online learning have been identified as a convenience, choice, of course, hedonic learning, ease of learning, customization, and personalization (Truong et al., 2017). While learners are aware of the advantages of online learning, they are equally aware of the risks of privacy that are associated with it (Schaik et al., 2017; Gogus and Saygn, 2019). Privacy protection is essential for learners because it fosters the intellectual development of society by providing an environment in which ideas can be nurtured and developed (Hughes, 2015). Online learning poses a greater privacy risk due to its ability to capture, store, and disseminate information at scale through technological means, as learners leave behind more personal details detailing their behavior and preferences during the online learning process. Consequently, online environments have become prospective targets of data breach attacks (Anwar, 2021). Studies have found that users’ perceptions of privacy risks and expectations of privacy protection are directly related to the continuity of courses, learning activities, and learning performance (May and George, 2011; Jim, 2021). Besides benefits and risks, studies showed that there was a significant correlation between the trust level of online services and disclosure intentions (Metzger and Flanagin, 2013; Fletcher and Park, 2017). Users tend to share personal information for the benefit of the overall process as long as they trust the shared environment and remain in control of their data. Moreover, security breaches that compromised the credibility of the system adversely affected students’ willingness to use online learning (Nurkhin, 2020). Thus, this study utilized the Privacy Calculus framework to examine the impact of online learning from three perspectives: perceived benefits, perceived risks, and perceived trust.

Prior research has provided useful evidence in explaining the success factors that influence learners’ willingness to use and adopt behavior in online learning from the benefit perspective. Extensive research has been conducted in the learning environment, social environment, learner’s perception experience, and learner’s characteristics (Alshurafat et al., 2021; Jiang et al., 2021; Li et al., 2021; Maheshwari, 2021; Punjani and Mahadevan, 2021).

In addition to the benefit perspective, some researchers have paid attention to the negative factors that cause learners to worry and hesitate in online learning and studying the impact assessment of online learning adoption willingness from the perspective of privacy. Through thematic analysis, Khlaif et al. (2021) revealed that infrastructure factors, cultural factors, digital inequality, and digital privacy threats influence student engagement in online learning. Zhai et al. (2020) conducted an investigation using the stimulus-organism-response (SOR) paradigm to examine how privacy concerns influence learners’ perceptions of knowledge hiding, thereby affecting their online collaboration. The results showed that two types of privacy concerns (abuse and unauthorized access to private data) affected students’ perceptions of knowledge hiding and negatively influenced their participation in collaborative learning online. Kim (2021) has conducted an insightful study examining the factors that motivate and hinder Reserve Officers’ Training Corps (ROTC) students from participating in online courses. Their results indicated that perceived usefulness and peer behavior directly affect participation intentions. Privacy and security concerns had a negative impact on perceived ease of participation, which is mediated by perceived usefulness and peer behavior. However, empirical research focusing on both benefits and privacy perspectives is still quite limited in the online education domain.

From the trust perspective, researchers mainly focused on collaborative learning and group interaction in online learning (Nam, 2014; Du et al., 2018). Researchers also quantified the perceived experience between privacy and trust to test the effectiveness of e-learning security mechanisms (Anwar and Greer, 2012). While some researchers have constructed theoretical frameworks for online learning related to benefits, privacy, and trust, most of the studies did not combine benefits and privacy. For example, Wang (2014) focused on a social-technical framework that includes credibility, design, instructor socio-communicative style, and privacy and security for the trustworthiness of an online course without considering its benefit. Therefore, this study intends to fill the gap by studying the relationships between learning benefit, privacy, and trust in online learning based on the privacy calculus theory. This will provide evidence to assist institutional decision-making to place emphasis on the services of online learning, promote a safe and trustworthy online learning environment, and enhance learners’ overall online learning experience.

### Benefit Perception

Benefit perception is a subjective evaluation of potential gains or favorable outcomes. According to Chen and Dubinsky (2003), benefit perception is the net gain obtained by consumers over the perceived cost to obtain certain expected benefits. Benefit perception can also be linked to the expectancy of success and task value in the expectancy-value theory (Loh, 2019; Poort et al., 2019). The expectancy-value theory holds that people’s motivation to choose a certain task is determined by their expectation of the possibility of success of the task and the value they attach to the task (Wigfield and Eccles, 2000). Eccles et al. (1983) identified the three main components associated with the theory: achievement value, intrinsic value, and utility value. Achievement value refers to the importance of doing a task well in terms of personal values, intrinsic value refers to the intrinsic enjoyment a person experiences from completing a task, and utility value refer to the usefulness of a task in helping people achieve other short-term or long-term goals. Chiu and Wang (2008) found that achievement value, utility value, and intrinsic value related to benefits are important predictors of individuals’ intention to continue using online learning. In the online learning environment, benefits include the convenience of acquiring knowledge, money and time savings provided by services, as well as the satisfaction and enjoyment obtained by students through online learning and interaction, including both material benefits and well-being benefits. Therefore, this study posits that the higher the students perceive the benefits brought by online learning, the higher their intention to learn from an online learning platform. The following hypothesis is proposed:

**H_1_**: Benefit perception (BP) is positively related to students’ willingness to use online learning.

### Privacy Perception

Privacy is defined as “the right of an individual, group, or organization to demand that they decide when, how, and to what extent information about them is communicated to others” (Westin, 1968). Privacy perception is the degree to which an individual perceives their right to control personal information. Privacy is violated when individuals do not have adequate control over the collection, storage, use, and disclosure of their personal information (Woodman et al., 1982). Privacy concern induces fears that information disclosure may cause undesired consequences (Xu et al., 2011a). Lwin et al. (2007) found that due to concerns about online privacy, users would use tools and techniques to protect the privacy and conceal information, such as using false information to disguise their identities. The stronger the user’s privacy perception, the more likely they are to realize the importance of information privacy, pay more attention to the security and protection of information privacy, and be more cautious when carrying out online activities. Therefore, the following hypothesis is proposed:

**H_2_**: Privacy perception (PP) is negatively related to students’ willingness to use online learning.

### Risk Perception

The concept of perceived risk was introduced by Bauer (1960) as the unexpected and uncertain outcomes that are typically unpleasant. Perceived risk has evolved from a two-dimensional construct of uncertainty and negative consequences (Brindley, 2005) to a multidimensional construct that includes financial, performance, physical, psychological, social, technological, communication, and time risks (Bertea and Bertea, 2011). In this study, risk perception is the students’ expected judgment of the worst possible outcome of personal information being excessively disclosed or subjected to cyber-attacks in the online learning platform. As online learning environments are exposed to constant security threats, risks, and attacks (Chen and He, 2013), it is necessary to assess how students perceive online learning risk. Mohamed et al. (2011) developed a scale to measure students’ risk perception of online education and found that performance risk, time-loss risk, psychological, and source risks were strong predictors of online learning intentions. A study by Lim and Zailani (2012) showed that perceived risks affect students’ intention to enroll in the online MBA program. In mobile learning, perceived risk had a significantly negative moderating effect on the relationship between performance expectancy and behavioral intention (Chao, 2019). Hence, when the students’ perceived risk exceeds its acceptable threshold, they may have concerns about continuing to use online learning. Therefore, the following hypothesis is proposed:

**H_3_**: Risk perception (RP) is negatively related to students’ willingness to use online learning.

### Trust Perception

Trust is defined as a mental state comprising of expectancy of a specific behavior from a trustee, belief that the expected behavior occurs, and willingness to take the risk for that belief (Anwar, 2021). Trust Perception is defined as “students’ perceptions about the reliability and trustworthiness of the system” (Arpaci, 2016). When users were faced with unfamiliar information systems, trust affected their decision whether to adopt (Gefen and Straub, 2003). Trust was found to be a significant predictor of behavioral intention in online learning (Harja et al., 2019). For instance, in online learning, trust must exceed a threshold level to accept assessment and recognition practices (Tereseviciene et al., 2020). Any security risk in online learning greatly affected students’ perception of the reliability and credibility of learning through the Internet. The attractiveness of online learning decreased, and the development of online learning was hindered (Adams and Blandford, 2003). Furthermore, trust was a precondition for self-disclosure that reduced the perceived risks involved in the disclosure of sensitive information (Anwar, 2021). Learners’ trust perception toward online learning is affected by data policy such as how sensitive data are shared and protected. For an instant, Kayali et al. (2019) investigated the adoption of cloud-based e-learning for delivering lectures and utilizing a learning management system and office application to facilitate the daily communication between students and lecturers, finding that trust in the cloud service providers affected user intention to adopt online learning. Therefore, this study believes that the stronger the students’ trust perception, the stronger their willingness to use online learning. Moreover, trust has an indirect effect on willingness to online learning through the mediating effect of risk perception. Therefore, the following hypotheses are proposed:

**H_4_:** Trust perception (TP) is positively related to students’ willingness to use online learning.

**H_5_:** Trust perception (TP) is negatively related to risk perception (RP).

**FIGURE 1:**
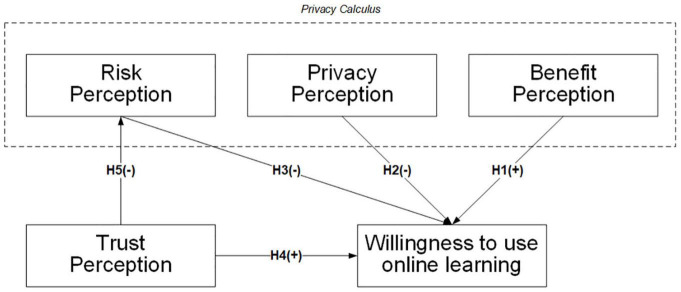
Privacy calculus and trust research model. +: positive effect, –: negative effect.

The research model is shown in Figure 1.

## Materials and Methods

### Participants

To test our proposed model, having learning experiences in online learning was the main requirement of the respondent. University students already have experience in using online learning and thus are ideal participants for this study. In this study, from March 9 to 11, 2020, data on the use of online learning were collected anonymously from undergraduates in a university in Hubei Province, China. A questionnaire was used as the primary data collection instrument, and copies were distributed online through QQ, WeChat, and Weibo. In a survey, 215 questionnaires were returned, and questionnaires with incomplete responses, straightening responses, or response time less than 40 s were removed. A total of 203 valid questionnaires were obtained, with a response rate of 94.4%. The respondents’ profile is presented in Table 1. The sample contained 45.32% males and 54.68% females.

**TABLE 1:** Respondents profile (*n* = 203).

Profile	Frequency	Percent (%)
**Gender**		
Males	92	45.32%
Females	111	54.68%
**Major**		
Science	113	55.67%
Liberal arts	49	24.14%
Engineering	36	17.73%
Physical education	5	2.46%
**Year of study**		
Freshman	53	26.11%
Sophomore	41	20.20%
Junior	47	23.15%
Senior	62	30.54%
**Online learning experience**		
Less than 1 year	95	46.80%
1–2 years	50	24.60%
2–3 years	34	16.70%
More than 3 years	24	11.80%
**Perceived frequency of privacy breaches**		
Very frequently	10	4.90%
Often	28	13.80%
Generally	72	35.50%
Occasionally	66	32.50%
Never	27	13.30%

### Instruments

The questionnaire survey intends to investigate the relationship between variables and to test the proposed hypotheses. The questionnaire was divided into two parts. The first part consisted of demographic information such as gender, major, year of study, online learning experience, and perceived frequency of privacy breaches. The second part of the questionnaire consisted of five dimensions measuring benefit perception, privacy perception, risk perception, trust perception, and willingness to use online learning.

As shown in Table 2, benefit perception adopted the learners’ perceived usefulness of the online learning platform developed by Lin and Wang (2012) and the perceived benefits of the intention to mobile applications by Wang et al. (2016). Privacy perception adopted the privacy perception security by Workman et al. (2008) and perceived ability to control information in privacy concerns with the use of the Internet by Dinev and Hart (2004). Risk perception adopted the risk perception from Pavlou (2003) consumption tendency and perceived risk of information disclosure of the location-aware marketing by Xu et al. (2011b). Trust perception adopted the consumers’ trust in online platforms from Grazioli and Jarvenpaa (2000). The willingness to use online learning adopted the Continued to Use Intention by Lin and Wang (2012). Overall, the five factors in the research model were measured with 15 closed-ended questions. A five-point Likert scale was used to measure respondents’ opinions, with five representing “strongly agree” and one representing “strongly disagree.”

**TABLE 2:** Measurement items.

Constructs	Items	Statements	Sources
Benefit Perception (BP)	BP1	I think the online learning platform is very convenient.	Lin and Wang, 2012; Wang et al., 2016
	BP2	I think online learning can save money.	
	BP3	I think the online learning platform can save time.	
Privacy Perception (PP)	PP1	I think of privacy as a right that I can control and use.	Dinev and Hart, 2004; Workman et al., 2008
	PP2	Controlling privacy is very important to me.	
	PP3	I think it’s very important to know how my personal information is being used.	
	PP4	When an online learning platform asks me to provide personal information, I need to weigh the risk.	
Risk Perception (RP)	RP1	I think there are risks in using online learning.	Pavlou, 2003; Xu et al., 2011b
	RP2	I think the use of online learning increases the risk of personal privacy breaches.	
	RP3	I am concerned about privacy breaches due to an attack on the online learning platform.	
Trust Perception (TP)	TP1	I think the vast majority of online learning is trustworthy.	Grazioli and Jarvenpaa, 2000
	TP2	I believe that online learning will fulfill its promise to protect personal privacy.	
	TP3	I believe that the online learning platform will not arbitrarily use my personal privacy information.	
Willingness to use online learning (WTL)	WTL1	I am willing to use online learning to learn.	Lin and Wang, 2012
	WTL2	I would like to recommend the online learning platform to my relatives and friends.	

## Results

A structural equation model (SEM) was used to analyze the research model and the correlation between the factors. Because structural equation modeling helps reveal linear relationships between observed and latent variables (MacCallum and Austin, 2000). In this section, factor analysis, reliability, and validity analysis were conducted to test the consistency of the measurement. AMOS software was then used to test the research hypotheses.

### Measurement Analysis

Exploratory factor analysis is used to determine the underlying structure for each construct. Factor loadings for each item should be greater than 0.5, indicating that all constructs have been discovered (Campbell and Fiske, 1959). As shown in Table 3, all factor loadings were greater than 0.6 and met the conditions.

**TABLE 3:** Factor analysis, construct reliability, and convergent validity.

Constructs	Items	Factor loading (>0.6)	Cronbach’s alpha (>0.7)	Composite reliability (CR > 0.7)	Average variance extracted (AVE > 0.5)
Benefit Perception (BP)	BP1	0.676	0.785	0.787	0.556
	BP2	0.881			
	BP3	0.659			
Privacy Perception (PP)	PP1	0.799	0.855	0.883	0.654
	PP2	0.876			
	PP3	0.812			
	PP4	0.742			
Risk Perception (RP)	RP1	0.833	0.804	0.845	0.645
	RP2	0.784			
	RP3	0.792			
Trust Perception (TP)	TP1	0.806	0.874	0.860	0.672
	TP2	0.834			
	TP3	0.819			
Willingness to use online learning (WTL)	WTL1	0.781	0.800	0.703	0.543
	WTL2	0.690			

For reliability analysis, Cronbach’s alpha and composite reliability were used to test the reliability of internal consistency. The measurement model is considered reliable when both Cronbach’s alpha (Field, 2013) and Composite reliability (Hair et al., 1998) values are greater than 0.7. As shown in Table 3, both Cronbach’s alpha and Composite reliability are greater than 0.7, indicating that each structure exhibits strong internal reliability.

For validity analysis, this study used convergent validity and discriminant validity for evaluation. To satisfy convergent validity, the Average Variance Extracted value should be 0.5 or higher (Hair et al., 1998). As shown in Table 3, the AVE values of the constructs were all greater than 0.5, meeting the condition of convergent validity. To demonstrate discriminant validity, the square root value of the AVE for each latent construct should be greater than the estimated correlation between these constructs (Fornell and Larcker, 1981). As shown in Table 4, the square root values of AVE for all constructs were higher than the correlation between these constructs, satisfying the discriminant validity condition.

**TABLE 4:** Correlation matrices and discriminant validity.

Construct	BP	PP	RP	TP	WTL
Benefit Perception (BP)	**0.75**				
Privacy Perception (PP)	−0.122	**0.81**			
Risk Perception (RP)	0.062	0.461	**0.80**		
Trust Perception (TP)	0.500	−0.223	−0.267	**0.82**	
Willingness to use online learning (WTL)	0.641	−0.208	−0.222	0.636	**0.74**

*Square roots of the AVE are presented as diagonal elements.*

### Structural Model Analysis

This study used a goodness-of-fit analysis to assess how well the proposed model fits the collected data. Then, the hypotheses proposed in the research model were tested. As described in Table 5, the results showed that χ^2^/df (2.592), AGFI (0.829), RMSEA (0.089), CFI (0.916), and IFI (0.917) were within the recommended range, and GFI (0.883), NFI (0.867), and TLI (0.871) were both greater than 0.85, which is acceptable. In summary, the values of these model fit indices, as shown in Table 6 confirm that the model has a reasonably good fit.

**TABLE 5:** Results of model fit indices.

Indices	Observed values	Recommended values	Sources
χ^2^/df	2.592	<5.00	Kline, 2011
GFI	0.883	>0.90	Bagozzi and Yi, 1988
AGFI	0.829	>0.80	Fornell and Larcker, 1981
RMSEA	0.089	<0.10	Hair et al., 1998
NFI	0.867	>0.90	Hair et al., 1998
TLI	0.871	>0.90	Bentler and Bonett, 1980
CFI	0.916	>0.90	Fornell and Larcker, 1981
IFI	0.917	>0.90	Keith and Jane, 2003

**FIGURE 2:**
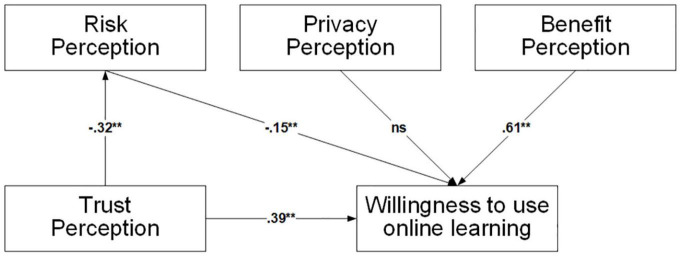
Results of hypothesis tests (*n* = 203). *p < 0.05, **p < 0.01.

**TABLE 6:** Results of hypothesis tests (*n* = 203).

		Standardized (β)			
Hypotheses		Total effect	Direct effect	Indirect effect	Supported
H1	BP→WTL	0.61**	0.61**	–	Yes
H2	PP→WTL	−0.02	−0.02	–	No
H3	RP→WTL	−0.15*	−0.15*	–	Yes
H4	TP→WTL	0.39**	0.34**	0.05**	Yes
H5	TP→RP	−0.32**	−0.32**	–	Yes

The hypothesis was tested based on the direct effect results, *p < 0.05, **p < 0.01.

To test hypotheses, a path analysis was performed on the hypothetical model. Figure 2 depicted the results of the analysis which showed that: (H1) benefit perception significantly affects willingness to use online learning (β = 0.61, *p* < 0.01); (H2) privacy perception’s path to the willingness to use online learning is not significant at *p* > 0.05; (H3) risk perception significantly affects willingness to use online learning (β = −0.15, *p* < 0.05); (H4) trust perception significantly affects willingness to use online learning (β = 0.34, *p* < 0.01); and (H5) trust perception significantly affects risk perception (β = −0.32, *p* < 0.01). Overall, the structural model explained 10.4% of risk perception and 82.6% of willingness to use online learning.

To further examine the impact of risk perception in trust perception and willingness to use online learning, the meditation test for the indirect impact suggests that trust perception’s effect on willingness to use online learning through risk perception (β = 0.05, 95% CI = 0.01–0.13) is significant. Table 6 showed the results of the direct effects, indirect effects, and the total effects among the variables by performing bootstrapping.

## Discussion

Based on the privacy calculus, this study develops a research model to understand the influence that benefit perception, privacy perception, risk perception, and trust perception have on the willingness to use online learning. We conducted a structural equation analysis to validate both the research model and the hypotheses. Results indicate that the benefit perception has a direct positive influence on students’ willingness to use online learning whereas the risk perception has a negative influence on students’ willingness to use online learning which is consistent with that of Chiu and Wang (2008). And the perceived benefits outweigh the perceived risks (Abduljawad et al., 2020). This result is consistent with the privacy calculus theory. When the perceived benefit outweighs the perceived risk, students are more likely to engage in online learning. Online learning provides students with the convenience of time and costs savings as well as learning modules that promote knowledge acquisition over potential risks. These results suggest that students may be motivated to use online learning if the perceived benefits are heightened.

The findings show that the risk perception negatively impacts students’ willingness to online learning which is consistent with the findings of Mohamed et al. (2011) where the four aspects of perceived risk were significantly and negatively correlated with intention to enroll in online learning. To provide personalization, online learning requires students to provide personal information, which students perceived to make them vulnerable to privacy risks. When students perceive the risks are high, they will tend to protect their privacy and reduce the behavior of endangering their privacy, which hinders their willingness to learn online. Therefore, to motivate students to use online learning, it is recommended that institutions should better understand students’ privacy fears and concerns. For example, they can promote certain security features of online learning to alleviate this concern, and publish and promote their data protection policy statements such as the General Data Protection Regulation (GDPR) and confidentiality options to reduce students’ insecurities (EDUCAUSE, 2021; Osterman, 2021).

Trust perception is found to have a positive significant effect on students’ willingness to use online learning which is consistent with the recommendation of Anwar and Greer (2012) and Anwar (2021) who advocated privacy-preserving reputation management (RM) to instill trust in online e-learning environments. The findings indicate that trust perception can positively influence students’ willingness to use online learning by reducing perceived risk which is consistent with the study of Hung and Wu (2012). Therefore institutions must ensure their online learning maintains good data protection reputations and trust mechanisms by adopting privacy-preserving technology (PPT) such as blockchain (Ullah et al., 2021) which will reduce the risk perception of students and motivate them to use online learning (Chang, 2021).

Interestingly, it is found that privacy perception is not affecting students’ willingness to use online learning. As indicated by this study, students are very concerned with privacy (*M* = 2.3), yet they are still open to using online learning (*M* = 2.5). This inconsistent behavior is often referred to as the “privacy paradox” (Barth and de Jong, 2017). There are various plausible explanations for such inconsistency. First, their trust in online learning and their need to obtain learning resources, student services, and social connections outweigh the privacy risks. Moreover, their use is motivated by the need to acquire knowledge in a timely and cost-effective manner to begin or continue learning. Additionally, although many students and teachers have expressed their appreciation for the importance of safety, the quality, and convenience of learning remain a significant part of what students and teachers expect (EDUCAUSE, 2021). Second, students may not be aware of the risks associated with the disclosure of information. The frequency with which students feel that their privacy has been leaked in the online learning platform (*M* = 3.35) is between “normal” and “occasionally.” In the process of online learning, the threat of cyber-attacks is often neglected or downplayed. Although some students may express their concerns, research showed that most students have limited ability to deal with data privacy issues because they lack the necessary knowledge and skills to handle these issues (Sideri et al., 2019). Dinev et al. (2015) revealed that individuals with fewer cognitive resources or with a heavier cognitive burden tend to perform low-effort processing. Low-effort processing is characterized by spontaneous reactions that lead to irrational behaviors that contradict the beliefs of the individual. The lack of transparency in the use of data, or the fact that they cannot prevent data breaches, may lead to users abandoning the protection of their personal information, resulting in a low motivation for users to take steps to secure their data.

### Research Implications

As one of the few studies to examine the impact of privacy security issues on online students, this study has a number of important implications. From a theoretical perspective, this study extends the privacy calculus theory into the area of online learning which enables us to develop a better understanding of how student perceptions of benefits, risks, and trust contribute to the willingness to use the online learning environment. The findings highlight the necessity for balancing personalization, privacy, and trust in the design of privacy and risk-aware online learning environments.

From a practical perspective, the research provides evidence and guidance to help online providers improve the safety concerns of online learning. For instance, providers can improve students’ and educators’ awareness of cyber security threats and promote the sound development of online learning security mechanisms. Likewise, online learning system developers should place a more significant emphasis on the security of their systems, along with the quality of control of their offerings and the technical capabilities of their systems. Online education providers have not been adequately prepared to handle network security issues. Many information technology support teams lack adequate human and financial resources to effectively manage network security (Osterman, 2021). Therefore, online learning providers should invest more in network security, solve the problem of information security worker shortages, and develop mature security measures. Online learning providers should ensure the confidentiality, integrity, and availability of data. Thus, students’ concerns regarding privacy and risk perceptions in relation to online learning systems are alleviated. The lack of transparency in what online learning is collected, how they are used, and how they benefit students, undermines trust in institutions’ ability to protect students’ data (EDUCAUSE, 2021). It is recommended that online learning providers implement a comprehensive data governance system to protect and manage student privacy so that students can have confidence in the security of their online learning environment and achieve a positive online learning experience.

### Limitations and Future Work

This study has some limitations. First, the current samples consist of only students from a single university in China. Since different provinces and universities provide different online learning environments, the samples need to be expanded to encompass different segments of the population to generalize the results. Second, the study was conducted which involved a completely online mode of learning rather than a blended learning approach. Thus, the results are relevant to this situation. Additional studies are needed to validate the different learning modes. Last, while the variance explaining students’ willingness to use an online learning platform is high, it is still possible that other variables were not considered, such as individual traits or personalities that are susceptible to risk. It may be possible to include such variables as part of an extended model in the future.

In this study, privacy perception did not seem to affect students’ willingness to engage in online learning, which may be explained by the privacy paradox. Additional research could clarify the extent to which privacy influences students’ willingness to use online learning by taking into account all moderators and individual factors, such as gender, age, education, personality, and learning level to arrive at a comprehensive privacy calculus model operate in the online learning context.

## Conclusion

Even though the use of online students’ data offers several benefits to students, including improved functionality and enhanced services, privacy and data security remain concerns for students. A privacy calculus theory is presented in this study to examine the factors that will assist online learning providers in addressing privacy protection concerns. The model was empirically tested using 203 data samples from a university in Wuhan, Hubei, China. Benefit perception and trust perception are the two factors that have the greatest effect on students’ willingness to learn online. It can be stated that the proposed model is useful for explaining students’ willingness to use online learning. These findings allow us to better understand what factors contribute to increased willingness to participate in online learning. Additionally, the findings can assist online learning providers and educational institutions in developing guidelines and policies designed to enhance online learning capabilities and quality of service.

## Data Availability Statement

The raw data supporting the conclusions of this article will be made available by the authors, without undue reservation.

## Ethics Statement

Ethical review and approval was not required for the study on human participants in accordance with the local legislation and institutional requirements. The patients/participants provided their written informed consent to participate in this study.

## Author Contributions

XJ contributed to the study design, theoretical basis, data analysis, and discussion of the manuscript. T-TG contributed to the discussion and conclusions of the manuscript and critically revised the manuscript. ML was responsible for the conception, data collection, and analysis of the manuscript. All authors contributed to the article and approved the submitted version.

## Conflict of Interest

The authors declare that the research was conducted in the absence of any commercial or financial relationships that could be construed as a potential conflict of interest.

## Publisher’s Note

All claims expressed in this article are solely those of the authors and do not necessarily represent those of their affiliated organizations, or those of the publisher, the editors and the reviewers. Any product that may be evaluated in this article, or claim that may be made by its manufacturer, is not guaranteed or endorsed by the publisher.
